# Fragmented understanding: exploring the practice and meaning of informed consent in clinical trials in Ho Chi Minh City, Vietnam

**DOI:** 10.1186/s12910-023-00884-2

**Published:** 2023-01-16

**Authors:** Yen Hong Thi Nguyen, Thuan Trong Dang, Ngoc Bao Hong Lam, Phuong Thanh Le, Phu Hoan Nguyen, Susan Bull, Evelyne Kestelyn, Jennifer Ilo Van Nuil

**Affiliations:** 1 Oxford University Clinical Research Unit, 764 Vo Van Kiet, Ward 1, District 5, Ho Chi Minh City, Vietnam; 2 Hospital of Tropical Diseases, Ho Chi Minh City, Vietnam; 3 The Ethox Centre, Nuffield Department of Population Health, University of Oxford, Oxford, UK; 4 Department of Psychological Medicine, Faculty of Medical and Health Sciences, University of Auckland, Auckland, New Zealand; 5 School of Medicine, Vietnam National University, Ho Chi Minh City, Vietnam; 6 Centre for Tropical Medicine and Global Health, Nuffield Department of Medicine, University of Oxford, Oxford, UK

**Keywords:** Informed consent, Clinical trial, Understanding, Motivations, Socio-cultural context, Inequality, Vietnam

## Abstract

**Background:**

The informed consent process in clinical trials has been extensively studied to inform the development processes which protect research participants and encourage their autonomy. However, ensuring a meaningful informed consent process is still of great concern in many research settings due to its complexity in practice and interwined socio-cultural factors.

**Objectives:**

This study explored the practices and meaning of the informed consent process in two clinial trials conducted by Oxford University Clinical Research Unit in collaboration with the Hospital for Tropical Diseases in Ho Chi Minh City, Vietnam.

**Methods:**

We used multiple data collection methods including direct observervations, in-depth interviews with study physicians and trial participants, review of informed consent documents from 2009 to 2018, and participant observation with patients’ family members. We recruited seven physicians and twenty-five trial participants into the study, of whom five physicians and thirteen trial participants completed in-depth interviews, and we held twenty-two direct observation sessions.

**Results:**

We use the concept “fragmented understanding” to describe the nuances of understanding about the consent process and unpack underlying reasons for differing understandings.

**Conclusions:**

Our findings show how practices of informed consent and different understanding of the trial information are shaped by trial participants’ characteristics and the socio-cultural context in which the trials take place.

## Introduction

For several decades, the informed consent process in biomedical research with human participants has been a matter of concern for both researchers and ethicists. Although components of valid consent including giving aquadate information, ensuring study participants’ understanding of information and their voluntariness in making decision have been clearly defined [1], the complexities of what comprises valid consent remain under discussion [2, 3]. Information provided during the consent process as well as the research participants’ comprehension of such information have been widely discussed as primary components of valid consent [4–6]. A major issue in the informed consent process is that study participants may lack of understanding of some study information when they consent to join a clinical trial [7, 8]. Research has also shown that socioeconomic and demographic characteristics of participants (e.g. advanced age, low level of education, and low socioeconomic status) can challenge the pratice of the informed consent and became barriers to understanding [7, 9, 10].

The varying interests of study sponsors and/or principal investigators, recruiting physicians, and participants often lead to multiple understandings, motivations, and potential conflicts of interest regarding informed consent [11–14]. For example, in the context of Vietnam, the use of the word “nghiên cứu” (research) in an information sheet may be legally required, but may cause confusion and raise fears in study participants and reduce the chance to recruit potential participants in research as the word “nghiên cứu” was associated negatively with “lab rats” or “guinea pigs” [13]. Explorations of study participants’ motivations to join research in low and middle income countries (LMICs) have found a range of reasons including “altruism, personal health benefits, access to healthcare, monetary benefit, knowledge, social support and trust” [11]. Further, discussions about understanding of research information, motivations and autonomy in clinical research address the concept of “therapeutic misconception” where participants think they are receiving standard healthcare instead of taking part in research activities, particularly when clinical research happens in hospitals [15–17]. Since then, ethicists have developed several concepts with an attempt to capture different shades of understanding and motivations for trial participation [18, 19]. One of those is the concept of “therapeutic optimism” which argues that study participants are not necessarily lacking understanding, but hope for the best personal outcome from the research in which they participate [18].

Finally, research has demonstrated that sociocultural and economic contexts strongly influence how informed consent is perceived and practiced; therefore those elements should be taken into account when practising informed consent [1, 20–22]. In LMICs, participants may develop knowledge about a clinical trial largely through informal dicussions about the trial among their community members rather than in informed consent sessions [23]. Moreover, participants’ trust in healthcare providers can lead to situations where participants, on varying levels, defer the decision making to the healthcare staff conducting research because of the nature of the relationship and/or severity of the health situation [24]. Previous research in Vietnam indicates that trust heavily influenced perceptions about research data sharing and research participation more generally [13, 25]. More broadly, many studies in low-resource settings identified challenges in achieving valid informed consent stemming from structural factors including poverty and unequal healthcare access [26, 27].

The informed consent process is a complex process interwoven with an intricate network of sociocultural and economic factors. To gain more knowledge of the practices and perceptions of informed consent in clinical trials, we conducted a qualitative study with physicians and trial participants in a hospital setting in Ho Chi Minh City. In this paper, we describe trial participants’ and study physicians’ reflections on understanding in consent sessions. We provide data on the practice of giving and obtaining consent from stakeholders, and show how the informed consent process and *fragmented understanding* were shaped by individual characteristics, motivations for conducting and participating in research and systemic factors. Our findings demonstrate that interpretation of universal research ethics guidelines in this context should be responsive to sociocultural factors and more work is needed to improve the quality of informed consent processes.

### Study context

Vietnam is a South-east Asian country with a population of 99 million people in 2022.^1^ Recognized as one of the fastest developing economies in the region, Vietnam has reduced its poverty rate to below 2% of total population.^2^ In 1992, Vietnam established social health insurance, which is regarded as the main method of public financing for health care. As of 2020, the social health insurance covers 90.85% of the population.^3^ However, out-of-pocket expenditure for health care remains high [28]. According to a human development report in 2020, although 95% of the Vietnamese population was literate, the mean years of schooling for the total population was only 8.3 years.^4^

The study took place in the Oxford University Clinical Research Unit (OUCRU) in collaboration with the Hospital for Tropical Diseases (HTD) in Ho Chi Minh City. HTD is the largest referral hospital for infectious diseases in Southern Vietnam. Since the start of the collaboration in 1991, OUCRU and HTD have been leading clinical and scientific research programmes with focuses on infectious diseases in the region. Studies in OUCRU follow international and national guidelines on ethical standards and procedures for biomedical research involving human subjects.

The study was embedded in two randomized clinical trials conducted by OUCRU and HTD. One was an out-patient clinical trial exploring the efficacy of shortened treatment for a chronic liver condition (Clinical Trial Registry number: 17IC4238) [29]. The other was an in-patient clinical trial which aimed to assess whether dexamethasone would improve prognosis patients with TB-meningitis (Clinical Trial Registry number: NCT03100786) [30]. Both clinical trials did not involve high-risk interventions, however some participants enrolled in the in-patient clinical trial were quite ill. Participants in the trials received drugs and examininations per trial protocol, as well as had travel costs reimbursed.

## Methods and analysis

From March 2019 to July 2019, we recruited potential participants from two clinical trials as explained above. Based on an estimation of the recruitment progress of the clinical trials, we designed the study to use purposive sampling for recruitment of up to 40 participants from a variety of backgrounds in order to maximize the range of experiences and perspectives. However, due to complications in recruitment (e.g. the trial recruitment was slower than anticipated), we ended up using convenience sampling and recruited only 25 patients in total. The study researcher (YHTN) waited in the waiting area for the clinical trials and approached potential study participants prior to them joining the trial’s informed consent sessions. We then explained the consent study and obtained consent from those who were interested to join in a less crowded section to protect their privacy as much as possible in this setting. Additionally, we collected information about study physicians’ experiences and perceptions of obtaining informed consent in the clinical trials. We obtained informed consent from all study physicians before talking with potential trial participants who had no information of whether the study physician joined the study or not. When a potential trial participant refused to take part in the study, we did not collect any data from them even if their study physician had already consented to join.

We conducted direct observations and documented the practice of informed consent in the two clinical trials. The study researcher (YHTN) observed the informed consent process and documented the process using handwritten notes and an observation guide. The researcher remained a sufficient distance from the session to avoid disturbing the process, with awareness of both physicians and potential trial participants. The observation guide included sections for descriptions related to duration, space and atmosphere, content of discussion and interactions between the physicians and the trial participants.

We did not assess the trial participants’ understanding of elements in the informed consent process through formal assessments of understanding. We used semi-structured interviews as the primary data collection method to explore the experiences and perceptions regarding consent seeking/giving, issues of comprehension of the information provided about clinical trials during the consent process, and reasons for joining or refusing to join a clinical trial. We planned to interview 13 to 15 patients and up to 5 physicians from the clinical trial teams, based on estimates from literature regarding data saturation in qualitative interviewing [31]. Interviews were audio recorded. For those who declined audio-recording, we took notes on the interview guide. All interviews occurred in a private setting in the hospital to protect the participants’ privacy. Recordings were transferred to a secured server within one to two hours after the interviews had finished. The interviews were transcribed verbatim and analysed in Vietnamese.

**Fig. 1:**
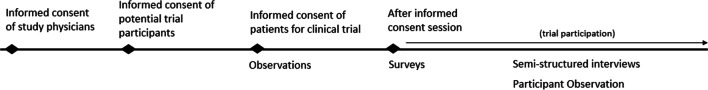
Data collection flowchart

We conducted twenty-two direct observation sessions of consent processes. Semi-structured interviews were conducted with thirteen patients and five physicians participating in the study. Of the thirteen interviews with trial participants, eleven were interviews with trial participants from the out-patient trial and two were with participants from the in-patient trial. We interviewed the trial participants from the out-patient clinical trial on their second or third study visit, about two to four weeks after the consent session. As the trial participants in the in-patient clinical trial had more severe symptoms, we waited until their recovery for the interview, often two or three weeks after the consent session (Fig. 1).

We conducted participant observation at the study sites and the spaces in which patient representatives and family members remained outside of the wards. Participant observation included documentation of the physical spaces, interactions among participants, and informal discussions about informed consent and clinical research more generally.

We also reviewed 26 informed consent forms and guidelines used for clinical trials in OUCRU from 2009 to 2018 to document the length, content and language used in the forms and to examine how the informed consent documents evolved over ten years. We developed the coding template using Karbwang et al.’s article [32] analyzing the essential elements for informed consent forms required by the Declaration of Helsinki, the International Conference on Harmonization (ICH) for Good Clinical Practice (GCP) and US Code of Federal Regulations. As the information sheet and the informed consent form used at OUCRU were based on different guidelines (i.e. the combination and adaptation of the ICH Guidelines for Clinical Good Practice [33], the guideline of Vietnam Ministry of Health [34] and of the Oxford University Research Ethics Committee [35]), we adapted the coding template to be suitable with the actual practice.

Data collected from observations, semi-structured interviews and fieldnotes were imported into NVivo 12 for coding. We created different codebooks for different types of data collection and study participants. Data were analysed thematically [36]. Coding was undertaken deductively based on the interview and observation guides, and inductively from the data. After the first round of coding, small codes were grouped into larger codes and we drew relationships between codes in the codebook and between the codebooks, and generated concepts and themes through an interpretative process.

### Study participants

We recruited seven physicians who undertook consent processes with prospective research participants, two from the out-patient trial and five from the in-patient trial. However, due to a tight schedule, one participant did not join any study activities. A total of twenty-five patients consented to take part in this study, including twenty-two potential participants in the out-patient clinical trial and three from the in-patient trial. Among the twenty-five trial participants in this study one declined to join the clinical trial after the informed consent session and two patients were not eligible to join the clinical trial. However, as their informed consent sessions were observed, they remained as our study participants. Trial participant ages ranged from 24 to 67 (median age 50). The educational level of the trial participants was quite low: 8/22 (36.4%) had at most five years of education and 7/22 (31.8%) had up to nine years of education. Only 5/22 (22.7%) had high school level education (up to 12 years) and only 2/22 (9.1%) had higher educational levels. Only five participants came from Ho Chi Minh City, while the rest came from different provinces in the south and the middle of Vietnam (Table 1 and Table 2).

**Table 1:** Demographic of study participants—trial participants and potential participants

Trial participants and potential participants	n (%)
*Gender*	
Male	9 (36)
Female	16 (64)
*Age*	
Up to 30	3 (12)
31–50	8 (32)
51–60	7 (28)
Above 60	7 (28)
*Educational level*	
No schooling	0/22 (0)
Grade 1—5	8/22 (36.4)
Grade 6—9	7/22 (31.8)
Grade 10—12	5/22 (22.7)
University or above	2/22 (9.1)
*Location*	
Ho Chi Minh City	5 (20)
Other provinces	20 (80)

**Table 2:** Demographic of study participants—study physicians

Study physicians	n (%)
*Gender*	
Male	3 (42.9)
Female	4 (57.1)
*Age*	
Up to 30	2 (28.6)
31–45	5 (71.4)
*Years of experiences in research*	
Under 1 year	1 (14.3)
1–5 years	5 (71.4)
6–10 years	0 (0)
Above 10 years	1 (14.3)
*Ward category*	
Out-patient ward	2 (28.6)
In-patient ward	5 (71.4)

## Results

In the following paragraphs, we first discuss the term “fragmented understanding” showing the nuances of understanding reflected by trial participants following the consent process. Then, we unpack the reasons and context surrounding fragmented understanding, including issues related to participants’ characteristics, the motivations of both study participants and study physicians, and the external factors going beyond individuals within this setting.

### What is fragmented understanding?

The term “fragmented understanding” describes trial participants’ different perceptions of research and clinical trial information. In the interviews, we explored which information about the studies was difficult to understand, with an expectation that the participants might have some knowledge of the trials in which they participated. As the interviews happened several weeks after their informed consent process, it was hard for trial participants to recall information provided during the consent process. In addition, many of the trial participants could not differentiate which consultation session with study physicians included the consent processes. We found that all interviewed participants understood that they were participating in research; however, apart from that, their understanding of the nature of research and of the information about the clinical trials varied. In the following paragraphs, we provide examples of these nuanced understandings.

We found that most participants hardly recalled any information when we asked them to reflect on the clinical trials in general, so we broke down the questions and prompted the participants about specific elements of the clinical trials. Although most of them could not explain the study in detail, they showed good understanding of several specific elements. Our data from observations demonstrated that those elements were either emphasized by the physicians or frequently asked about by the potential participants during the informed consent process. For example in the consent process with one participant, the physician repeated the information about the study procedure to make sure the participant understood her responsibility when she joined the study. The physician also repeated information about the side effects because the participant kept asking about that.

When we asked the participants in the out-patient trial about what they knew about the study, many of them started with the physicians’ emphasis on their study responsibilities.

“The physician told me to follow the treatment schedule. If I agreed to join the study, I would have to follow it. If I could not do it, I should have given this chance to another patient. I could not stop the treatment in the middle because it may worsen the disease. If I agreed to join the study, I would have to follow the physician’s procedure. I thought about that carefully and I agreed to join.” (Participant 11)

This example illustrates that participants’ understanding of study requirements was influenced by study physicians’ perceptions of critical aspects of the clinical trial. We also observed that the trial participants often recalled information about risks, benefits and interventions in case of injury in our conversations about their consent process. Study benefits were the best understood aspect of the study; most participants could point out the benefits that they received from their participation, even when they had limited understanding of other elements. While many participants in the out-patient trial showed good understanding about the study risks and side effects following the consent process and their experiences in the study; some participants did not have exact memories of those elements but they could recall that the study side effects were mild and uncommon and they accepted them as unavoidable risks of any type of medication they may take. The participants’ knowledge and experiences of the study benefits and the mild risks enhanced their confidence in continuing their participation in the study.

“I felt okay, although I had some sleep deprivation. But I do not have the side effects such as vomiting or dizziness like the physician said. I didn’t have those symptoms, so I determined to join [stay in] the study.” (Participant 07)

In the in-patient trial, one participant had difficulty recalling this information and misunderstood what side effects might occur, although information about side effects was of importance in the consent process, based on our observation of the session.

“I remembered that one of the two medicines used for this [disease] – I am not sure which one – will cause diabetes. I am not sure if I remembered well.” (Participant 15)

In contrast, study procedures and the study purpose were the most challenging for participants to recall and explain. Although many participants in the out-patient clinical trial insisted that they had understood the information provided during the consent process, they failed to recall it when interviewed.

“The physician told me all information [about the study]. She explained very carefully but I do not remember anything. I only understood it at that time.” (Participant 10)

We received similar responses from participants from the in-patient trial. When asked specifically about randomization and placebo use, which are important elements of a randomized controlled trial, the participants seemed to be skeptical about those terms.

“The physician probably talked about that, but I do not remember them.” (Participant 22)

“I have never heard about that [randomization and double-blindness].” (Participant 15)

Participants’ limited understandings of these aspects of research were also recognized by the study physicians who obtained their informed consent.

“I don’t think the patients understood what we were doing [study procedure]. They might want to know when we started to use the study drug or so.” (Study physician 05)

Lastly, misunderstandings about the nature of the research was often evident amongst research participants. The participants often talked about the research in terms of a guaranteed cure and free treatment. In the out-patient trial, although the study physician explained about the nature of research at the beginning of every consent session, many patients thought they were receiving specialised treatment and were confident about being cured after participating in the research; therefore, they felt grateful for being chosen in the study.

“The physician checked me very carefully so that they could treat me. […] I am so happy. I am happy to participate in this group [study], so that I can be cured.” (Participant 12)

Additionally, this patient misunderstood not just the reality of research, but also the role of confidentiality. She thought that it was her responsibility to keep the study information confidential but not the research team’s responsibility. In the interview, she asked the researcher if she could talk about the trial with her friends and relatives. Interestingly, other participants had similar misunderstandings about this term.

“I want to ask you a question. Now I am in the study; if someone asks me, should I tell them the truth? […] Should I let them know about this [trial participation]? Does the physician want it to be revealed?” (Participant 12)

Fragmented understanding is the term we use to describe the situation in which the trial participants showed varying degrees of understanding or misunderstanding of trial information. While partial understanding often reflects information perceived to be most important by the researchers and the participants, areas of no understanding and misunderstanding demonstrate that some core information of the study was unclear. In the next section, we will unpack the causes of fragmented understanding in our context.

### What is behind fragmented understanding?

Fragmented understanding stems from a complex network of individual and structural factors. In this section, we will discuss the factors contributing to fragmented understanding, including those relating to characteristics of the trial participants, motivations of both the participants and the physicians and broader systems in which the clinical trials took place.

### Characteristics of the participants

Trial participants’ characteristics, including older age, literacy level and health status during the informed consent process, had a significant impact on their understanding and recall of the study information. During the interviews, many older participants admitted that it was difficult for them to remember the study information due to their age.

“I don’t remember [the study information]. I am old now. I forget things. I don’t remember.” (Participant 10)

The physicians revealed that older people absorbed the information more slowly, which often caused great challenges for them during the consent process. Therefore, flexibility and creativity in the consent process were critical for physicians.

“It normally takes some effort to explain something to senior people. You must repeat the information over and over or explain it in different ways. When I obtained informed consent from senior patients, I often invited a family member to join the session. The patient signed the informed consent form; the family member listened to us. This person could explain the information in the way the patient might understand, while we couldn’t.” (Study physician 05)

The involvement of family members in the consent process, including having the family member help with interpreting the information into more familiar language, was a strategy that the physicians used to overcome some of the challenges arising when explaining the research to participants with low literacy and/or older age. The anxiety and shock that the participants and their family members had when they heard about the diagnosis of disease for the first time also impacted their capacity to understand the study.

“They were not in their best mental state, so it would be hard for them to acknowledge the ideas.” (Study physician 03)

While study physicians struggled to explain the study to some of their senior and low-literacy participants, additional participants found that the study information was too complicated for them to understand because of the specific knowledge it conveyed. In some cases participants decided to join the study without understanding some aspects.

“[…] If you tell me more about the study, I will still not understand it. Whatever you do, I will not understand it. We just know medicines can cure us. We don’t care about what [is] inside it. I can only understand it [the study] at my limit, I cannot understand it at your level, because this is your expertise.” (Participant 06)

In some cases, challenges with comprehending the study information were demotivating and participants found it difficult to maintain their attention throughout the consent process, despite the study team’s effort to explain the research. Researchers and participants acknowledged that trial participants’ characteristics including age, literacy level and health conditions affected the participants’ capacity to take in and understand the trial information.

### Motivations of the participants and study physicians

Different motivations of both study physicians and participants during the consent process contributed greatly to fragmented understanding. Some physicians participating in the study emphasized the recruitment purpose of the process. A physician from the in-patient clinical trial viewed his informed consent process was successful when the participants agreed to participate into the study, regardless of their level of understanding.

“My informed consent process was what I wanted it to be because I wanted to enroll patients into the study and most of them agreed to do so. […]. The patients understood and agreed to join the study; but their level of understanding did not seem to be in line with the definition of informed consent.” (Study physician 03)

When the physicians stressed recruitment as the focus of informed consent, they overlooked their responsibility to make the study understandable to all participants. Additionally, fragmented understanding happened not only when the physicians focused on recruitment rates, but also when the participants were very motivated to take part in research. There were many motivations for participants to join the studies, including existing financial constraints leading to hopes that research participation would enable better access to effective treatment; trust in the healthcare workers and the research based-hospital; and altruism for helping other patients in the future.

Some participants from the out-patient clinical trial had suffered from the chronic liver disease for a decade or more. Many had lost hope of being cured because they could not afford effective treatment. A treatment course of three to six months could be around ten to thirty times higher than the national average monthly income (in addition to the high cost of monitoring viral loads before, during and after treatment). In some cases participants faced lengthy and burdensome trips to the hospital for liver function maintenance medicines every few months. Hence, the clinical trial appeared to be a life saver and joining the trial was seen to be their only option to access treatment and be cured.

“I thought as long as I received the treatment, I would be cured without paying any fee. I don’t have money to treat the disease. I simply thought so, I did not think much.” (Participant 06)

The in-patient trial participants expressed the same thoughts when they were asked about their motivations for participation. Although the physicians were open about the nature of research, the hope for a cure was so great that the participants tended to cast away their doubts about the trial or become overconfident with hope of being disease-free.

“I am afraid [of joining the trial], but the viruses are already in my body. Therefore, I wanted to try this time. Maybe the medicine will work and I will be cured. I concerned about it a lot, but I decided to join the study.” (Participant 17)

Although most of the participants we interviewed viewed it as important to thoroughly understand the study before making decisions, some found information about the clinical trial was not important for their decision making as long as they received the treatment.

“It [the understanding level of trial information] did not affect me when I made the decision to participate the study, as we had to consider our situation. I told my wife: “I would accept everything”." (Participant 06)

Additionally, trust in the healthcare workers and the hospital where the clinical trials took place established a rationale for participation.

“I signed [the informed consent form] because I trusted them [the physicians]. I told my husband that the physicians have never been wrong; we should have not doubted if we followed them.” (Participant 22)

The genuine trust that some participants had in the study physicians led to their research participation with little consideration or understanding of the study information. This trust also created a dilemma for the physicians. On the one hand, a study physician found it challenging when her patients entrusted her to make decisions about their research participation. On the other hand, trust was recognized by the physicians as a critical element of the informed consent process so that they could better engage with the patients and improve the quality of informed consent.

“You have to build trust with the patients and then, explain the study clearly to them. Give them clear responses for their questions.” (Study physician 07)

In addition to trust in physicians, trust in the hospital and its collaboration with a foreign institution was often mentioned as a motivation for participation by the trial participants. Some of them emphasized the international involvement in the study made it more reliable.

“I felt reassured that I would receive good medicines. The fact that this study is conducted by an American university gives me a lot of assurance.” (Participant 15)

Although this participant mistook the origin of OUCRU, the foreign collaboration added another level of reassurance for her to join the study. With concerns of the medicine quality in the national market, she believed that a study conducted by an institution from a high income country would supply trusted medicines.

Furthermore, the participants often mentioned their empathy for other patients and a hope to contribute to a common good when they talked about motivation for joining the studies.

“I wish I would be cured, and the others too. The study may help more people with the disease to be treated because I saw so many patients out there.” (Participant 11)

### Beyond individuals

We found different systemic issues beyond individual characteristics and practice also caused fragmented understanding. Those included incomprehensible informed consent forms, the perceptions of informed consent as a legal process, and the culture of hierachical physician–patient relationships.

The informed consent forms were criticised as being too long and too complex for people with low literacy, by both physicians and trial participants. From the review of the information sheets and consent forms of clinical trials conducted in OUCRU from 2009 to 2018, we found that the information sheets included over twenty-five items and both forms were created in English language by the research team and translated into Vietnamese language for recruitment. The physicians who obtained informed consent from the potential participants were not involved in developing the consent documents. We identified that the biggest challenge in translation was to convey the meaning of complex concepts in English in simple Vietnamese. A strict translation might lead to confusion and even incomprehension. Although ethical guidelines highlight the importance of language in informed consent forms being non-technical and understandable for participants, their representatives and potential witnesses, there was no guideline for translating jargon into lay language. One example that arose from our interviews showed that many participants did not understand the term “confidentiality” (bảo mật) in Vietnamese; which led to some confusion about whose responsibility it was to keep the participants’ information confidential, as discussed above. The complex informed consent form was also a result of the perception of informed consent as a legal process. Most physicians in the study mentioned that informed consent had its legal function to protect both sides in conflicts; therefore, it should contain all possible details. In the context where most study participants did not finish elementrary level education, thorough understanding of the long and complicated informed consent form seems unrealistic.

Data from the study demonstrated that the cultural background shaping the hierarchical relationship between physician/study physician and patient/research participant was one of many contributors to fragmented understanding. Some physicians found it a challenge when their participants were too shy to admit that they did not remember or did not understand the study information. At the same time, many participants were afraid to disturb or to annoy the physicians with further questions, but still wanted the “treatment” so they agreed to join the study without thorough understanding. In many cases, the information sheet and consent form were kept and reviewed by participants as they sought to understand more about the study during their trial journey, even though verbal consent was provided and the study physicans were available to address their concern.

“Gradually, I understood [the study information]. For some information I could not understand, I just kept reading it, word by word. Slowly, I understood it in the end. But after that, I did not remember it.” (Participant 10)

The complex language in the consent form along with a hesitation to ask for more explanation about the study prevented participants accessing and understanding some information about the study.

## Discussion

Our findings suggest that the practice of informed consent processes and study participants’ fragmented understanding in this context stemmed from individual to sociocultural and systemic factors.

Trial participants’ old age, illiteracy and poor health at the time of consenting challenged their understanding of the study information. Nguyen and colleagues found that participants with low education level showed reduced understanding of the nature of the study, placebo, randomization and freedom to withdraw [7]. The study also revealed that study participants were aware of risks and side effects, but unable to name at least one risk. We found considerable similarity in results presenting that study participants showed some understanding of the research, without understanding all of the information. In our context, information related to risks and side effects appeared to be of most interest and importance for research participants. We could not assess whether the participants comprehended the trial information at the time of consent because the interviews were conducted a few weeks after consenting to the study, but we did identify challenges with recalling information. Even though recall and understanding are different concepts, and not being able to recall all detailed information does not necessarly mean not understanding at enrolment, the informed consent process should ideally be a continuous process throughout research [37]. We believe it is crucial to repeat key information at each visit and to offer research participants opportunities for reconsidering their participation during the trials. Further larger scale studies on assessing trial understanding in informed consent sessions and during research might be necessary in our context. There have been a great number of discussions on what is a valid informed consent, how much information is considered sufficient, and what information should be prioritized in the informed consent process [21, 32, 38]. Most authors agreed that potential research participants must acquire general ideas of research purposes, research procedures, participants’ responsibilities in research, their possibility of withdrawal and potential risks [38–40]. Moreover, disclosure of information should also be specifically tailored to local context and information that is of potential participants’ interests [21, 22, 38].

Understanding of the culture in which the informed consent process takes place is crucial [41–45]. Fan [45] demonstrated the fundamental differences of Western and East Asian bioethics by explaining the concept of autonomy in both perspectives which showed that Western principles of autonomy focused on self-determination, while the East Asian principles stressed family-determination and hamornious dependence. In Asian and African contexts, family members often play an important role in shared decision-making; therefore, it is culturally sensitive to involve a family member in the process [42]. We also found the practice of involving family members in the informed consent process to maximise chances of understanding in our context was logistically and culturally appropriate.

Trust in healthcare workers, in the hospital hosting the clinical trials and in an international collaboration was a significant element contributing to decision-making in our setting: participants decided to join the studies because they trusted that physicians would do no harm to them and at times they completely deferred to physicians when making decisions. Such trust motivated participants to participate in clinical trials, often without full understanding of the study information. Our findings are in line with previous studies on stakeholders’ perceptions of data sharing and consent in the same context [13, 25]. These studies showed that study participants made decisions based on high levels of trust, rather than based on their own consideration of the provided information. Studies in other settings also identified trust as a central element in health research [11, 46, 47] and arguments have been made that when trusting heathcare providers and healthcare institutions, study participants made themselves vulnerable in research [48, 49]. We echo similar reflections from our context in which participants entrusted their well-being to the healthcare workers and the hospital, showed their desperation for good healthcare that they deserved, making decisions with fragmented understanding of what might happen to them during research.

Access to healthcare was reported as the top motivation for participation in 42 out of 94 reviewed articles on motivations for research participation from LMICs [11]. Limited access to treatment due to financial difficulties was also the most mentioned motivation for clinial trial participation in this context. In Vietnam, although social health insurance exists with the aim to enhance the accessibility to healthcare for people living in poverty, informal workers face significant barriers to healthcare access due to the poor quality of primary healthcare services offered to insured patients and complex paperwork and referral procedures [28, 50]. Lack of support from the social security system, poverty and unequal access to quality healthcare services may result in clinical trial participation being the only feasible choice for participants to receive treatment. Participation in clinical trials for access to better healthcare is not necessarily a bad choice; however it may be a significant motivation that impacts patients’ capacity to make voluntary decisions [1].

In addition, the hierarchial relationship between patients and physicians orginating from the traditional social structure challenges attempts to meaningfully communicate during consent processes. Asking questions or requesting more information about health concerns is not the norm for Vietnamese patients in healthcare settings [51]. These social and cultural factors challenge the attempt to undertake meaningful informed consent and shape the choices of research participants, at times resulting in an “empty choice” [27]. Cases in which choices for research participation are defined and limited by the broader healthcare system have been reported across limited-resource settings. Van Nuil and colleagues reported a vignette in which study participants decided to take part in a study before receiving study information and argued that this fact revealed deeper social injustice and inequalities in Rwanda rather than problems in informed consent itself [23]. Critiques haven been raised that standard practice guidelines emphasize the disclosure of information but forget the quality of the decision-making process [52]; therefore the practice falsifies the ideas of informed consent which is supposed to be about understanding and informed choices to a legal process [38]. In our context, although many standard procedures have been in place to protect trial participants in research, we have found in this study that a standard version might not fit all circumstances. At the time of writing up this study, only a few studies had been conducted on informed consent for clinical trials in OUCRU and HTD [13]. Although suggestions for improving understanding of trial information have been discussed in other contexts[53], there had been no wide-scale intervention implemented to date towards improving the consent process in clinical research at the study site in OUCRU. However, the study physicians would often came up with solutions based on their experiences (e.g. inviting family members to the informed consent process). We found that there was little systemic support for researchers to obtain meaningful informed consent and recognised that improving the quality of informed consent practice is the collective effort of the study team and the institution conducting the trial, not just the physicians who are obtaining it.

### Recommendations

As this study showed the participants’ challenges of reading the information sheets and the informed consent forms, we conducted an engagement project to redesign the documents with pictures and larger font size and tested with the community advisory board. The board members found the revised forms positively improved their ability to read the forms and enhanced their understanding of the forms. Besides, strategies to improve the informed consent process and the decision-making process have been proposed in many studies. Schenker and colleagues [54] reported that additional written information, use of audiovisual and multimedia tools, extended discussions and test/feedback techniques improved the study participants’ comprehension, especially their understanding about risks and procedures. An adapted informed consent form developed with systematic self-reported information needs [55] and with contribution from different expert groups and tested by target audiences [53] was proved to improve the understanding of study participation. Moulton et al. [56] proposed a shared decision making strategy focused on being transparent about competing interests of stakeholders involved in the study and the alignment of patients’ goals and values with their decision of research participation. Moreover, being open about clinical research with the general public is also a method for improving quality of informed consent [57]. We, therefore, suggest further studies in OUCRU to explore effective and context focused methods to better engage and communicate with study participants about research and their participation in research.

## Limitations

The study had several limitations. First, the process of recruitment for this study often confused trial participants because we had to gain consent prior to the trial’s consent process, while many participants were not aware of what clinial trials are and what choice to participate would be offered to them. The idea of informed consent was abstract for trial participants who had not been through the process before. Therefore, we had to actively refuse to include a few number of participants who were interested to join the consent study but did not understand the study purposes after we explained it to them. Second, we could not enrol the planned number of participants from the in-patient trial because most patients admitted to the hospital were too unwell to participate in the consent study. The low number of participants from the in-patient clinical trial limited our research into their experiences and perceptions of informed consent. Finally, informed consent should be an on-going process, but we only observed the first informed consent session. This limitation prevented us from determining what information was provided in conversations during study participation. The fact that the trial participants could not recall their memories of the informed consent process also limited our analysis of their understanding of the study information to some extent. However, as explained earlier, our original aim was not to assess participants’ understanding of clinical trial information; we employed qualitative method to explore the perceptions and practices of the informed consent process. The method allowed us to have in-depth understanding of how the trial information was understood and misunderstood and how decisions were made despite their fragmentation of understanding.

## Conclusion

In this study, we explored the experiences and perceptions of study physicians and trial participants about the informed consent process in a clinical trial setting and demonstrated how individual and sociocultural factors shape the practices of informed consent and fragmented understanding of trial information. Characteristics of participants, motivations of participants and physicians for joining and conducting clinical trials, and structural factors including complex informed consent forms, barriers to healthcare, and hierarchical relationships between physicians and patients contributed greatly to fragmented understanding. When seeking to improve consent practices it is consquently necessary not just to provide resources and support to research teams, but also to recognize and appropriately respond to the impacts of structural elements on consent processes and understanding of research including improving the quality of healthcare and the effectiveness of social health insurance, strengthening the healthcare system and reducing inequality in healthcare.

## Data Availability

The datasets used and/or analysed during the current study are available from the corresponding author on reasonable request.

## Abbreviations

OUCRU: Oxford University Clinical Research Unit
HTD: Hospital of Tropical Diseases

## Footnotes

1 United Nations Population Fund. (2022). Vietnam Overview. Retrieved July 2022, from https://www.unfpa.org/data/world-population/VN

2 The World Bank in Vietnam. (2022). Overview. Retrieved July 2022, from https://www.worldbank.org/en/country/vietnam/overview

3 Vietnam Social Security. (2021). Possible aim for full population coverage of national health insurance (Bảo hiểm y tế là mục tiêu khả thi, hoàn toàn có thể đạt được). Retrieved July 2022, from https://baohiemxahoi.gov.vn/tintuc/Pages/linh-vuc-bao-hiem-y-te.aspx?CateID=169&ItemID=17426

4 Open Development Vietnam. (2022). Human Development Indicators – 2020 – Vietnam. Retrieved December 2022, from https://data.vietnam.opendevelopmentmekong.net/en/dataset/cac-ch-s-phat-tri-n-con-ngu-i-vi-t-nam/resource/26869f5d-fe89-419a-bf0c-8087c4a3aaba
